# Immune Modulation Through Stereotactic Radiotherapy: The Role of TBX21, GATA-3, FoxP3, and RORɣt

**DOI:** 10.3390/medicina61040717

**Published:** 2025-04-13

**Authors:** Aybala Nur Ucgul, Huseyin Bora, Gizem Yaz Aydin, Ozlem Gulbahar, Ummu Habibe Koken

**Affiliations:** 1 Department of Radiation Oncology, Gulhane Research and Training Hospital, Ankara 06010, Turkey; 2 Department of Radiation Oncology, Faculty of Medicine, Gazi University, Ankara 06830, Turkey; 3 Department of Medical Biochemistry, Faculty of Medicine, Gazi University, Ankara 06830, Turkey

**Keywords:** stereotactic body radiotherapy, immunology, radiobiology, immunotherapy

## Abstract

*Background and Objectives*: Stereotactic radiotherapy enhances local tumor control by delivering high doses directly to the tumor. It is thought to activate the immune system via T-cells, possibly creating a systemic response. This study aims to evaluate stereotactic body radiotherapy’s (SBRT) impact on the immune system by measuring T-cell transcription factors, such as TBX21, GATA-3, FoxP3, and RORɣt. *Materials and Methods*: Peripheral blood samples were collected from 103 patients before SBRT and from 66 patients two months post-treatment. We measured transcription factors TBX21, GATA-3, FOXP3, and RORγt using ELISA, and performed a complete blood count and C-reactive protein analysis to rule out infections. Statistical analyses included paired *t*-tests and correlation analyses to assess changes before and after treatment. *Results*: Post-treatment, significant reductions were observed in TBX21 (Th1), GATA-3 (Th2), and FOXP3 (Treg), while RORɣt (Th17) remained stable but trended higher in lung cancer patients. No correlations were found with demographic factors. However, TBX21 levels were significantly related to the planning target volume (PTV) and biologically effective dose (BED10) in the lung region. Larger PTVs (≥16.5 cc) and higher BED10 doses (≥100 Gy) were linked to smaller reductions in TBX21 (*p* = 0.008, *p* = 0.04) and increased RORɣt levels (*p* = 0.01). *Conclusions*: Stereotactic radiotherapy reduces immunosuppressive markers like FOXP3 and GATA-3, indicating its potential to boost immune activation by suppressing Treg and Th2 cells. Larger target volumes and higher BED10 values may enhance Th1 responses through TBX21. These findings suggest that SBRT activates the immune system, and its combination with immunotherapy could be promising.

## 1. Introduction

Stereotactic body radiotherapy (SBRT) has become a standard treatment modality for various primary and metastatic tumors. Its ability to precisely deliver high radiation doses while sparing surrounding healthy tissue has revolutionized cancer care [1,2]. Beyond its local effects, increasing evidence suggests that SBRT can induce systemic immune responses, potentially enhancing its therapeutic efficacy [3]. The “abscopal effect”, a term first introduced by Mole in 1953, describes the phenomenon where radiation-induced tumor regression occurs outside the irradiated field, mediated by systemic immune activation. This effect highlights the potential of SBRT to function not only as a local treatment but also as an immunomodulatory agent capable of enhancing systemic anti-tumor immunity [4].

T-cell transcription factors play a crucial role in mediating immune responses to SBRT. These factors, which can be classified as master regulators or signal transducer and activator of transcription (STAT) proteins, control the differentiation and function of T-helper cells. Specifically, T-box transcription factor 21 (TBX21) and STAT4 are associated with Th1 cells, GATA binding protein-3 (GATA-3) and STAT5 with Th2 cells, RAR-Related Orphan Receptor Gamma (RORɣt) and STAT3 with Th17 cells, and Forkhead box P3 (FoxP3) and STAT5 with Treg cells [5]. These master regulators influence the balance between pro-inflammatory and immunosuppressive pathways, which is essential for understanding SBRT immunological outcomes. TBX21 and RORɣt are linked to immune activation, while GATA-3 and FoxP3 contribute to immune suppression.

These specific transcription factors have been used in various studies to assess prognosis in tumor specimens, rather than focusing on the immune response to SBRT [6,7,8,9]. In the trials examining the immune response to SBRT, CD4+ and CD8+ T cells were the primary markers employed [10,11,12]. However, the use of these markers to evaluate the immune response to SBRT is limited in the current literature [12,13,14].

A prospective study involving 89 early-stage non-small cell lung cancer patients demonstrated that SBRT increased CD4+ and CD8+ T-cell levels and significantly changed transcription factors such as TBX21, GATA-3, and FOXP3. These findings suggest that SBRT may enhance Th1 activity while reducing Treg-mediated immunosuppression. However, no significant correlation was found between these immune changes and dosimetric parameters such as mean lung dose [14].

Most studies investigating the immune effects of SBRT have focused on specific cancer types, particularly non-small cell lung cancer and liver metastases [6,10,11,14]. A significant limitation of these studies is the lack of comprehensive treatment data, including information on chemotherapy, immunotherapy, and detailed dosimetric parameters. These omissions limit the comprehensive understanding of how SBRT modulates immune responses and restrict its integration into multimodal treatment strategies. Therefore, understanding the effects SBRT on the immune system in different types of cancer is important for the development of concurrent therapies, especially immunotherapies.

This study aims to address these limitations by evaluating the impact of SBRT on critical T-cell transcription factors, including TBX21, GATA-3, RORɣt, and FoxP3, in a diverse patient cohort. Additionally, we investigate the role of dosimetric parameters and prior treatment histories in shaping immune responses. This approach provides a more holistic understanding of the immunomodulatory effects of SBRT. Our findings may offer new insights into the immunomodulatory effects of SBRT and its potential integration with immunotherapy.

## 2. Materials and Methods

The study protocol was approved by our local ethics committee. The inclusion criteria comprised individuals aged between 18 and 90 years, with eligibility for SBRT for any indication. Patients with a history of autoimmune disease (e.g., rheumatoid arthritis, systemic lupus erythematosus), secondary cancer, bone marrow or organ transplantation were excluded from the study. Also, the study excluded steroid users from the commencement of treatment until the second blood collection. Written informed consent was obtained from all participants by the Helsinki Declaration.

### 2.1. Treatment Protocols

SBRT was delivered using a linear accelerator (Truebeam, Varian Medical Systems, Palo Alto, CA, USA) with volumetric-modulated arc therapy. The total dose and fractionation were selected based on the location of the tumor, its volume and the proximity of the organs at risk. Most patients in this study presented with brain metastases, primary lung tumors or lung metastases, and vertebral metastases. A smaller group of patients had liver or adrenal gland metastases, as well as glomus tumors. All treatments were performed by the departmental protocols. Lung tumors were typically treated with a total dose of 50–54 Gy, delivered in 3–5 fractions [15,16], while brain metastases received either 20–24 Gy in a single fraction or 27–30 Gy in 3–5 fractions [17,18]. For bone metastases, doses ranged from 24 to 35 Gy and were delivered over 3–5 fractions [19].

### 2.2. Study Flow

**Figure 1:**
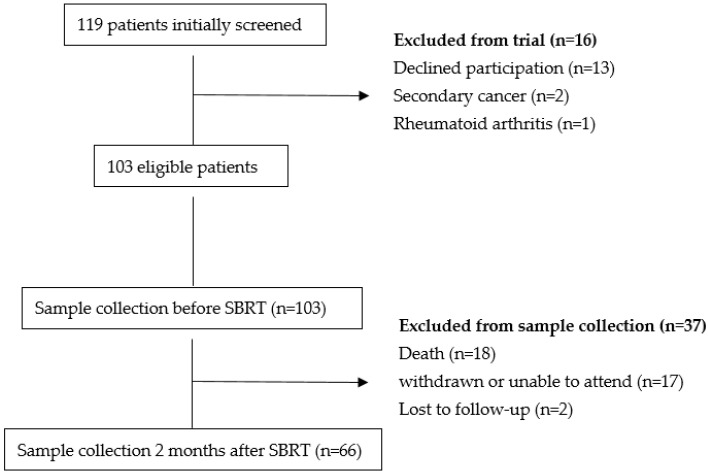
Flow Chart SBRT-Stereotactic Body Radiotherapy.

A total of 103 patients were included in the study before undergoing treatment. Initially, blood samples were collected from all 103 patients. However, during the follow-up period, 18 patients died, 2 were lost to follow-up, and 17 patients were unable to attend due to logistical or emotional reasons. As a result, post-treatment blood samples were collected from 66 patients two months after therapy (Figure 1).

### 2.3. Serum Transcription Factor Measurements

Serum levels of the transcription factors TBX21, GATA-3, FOXP3, and RORɣt were measured using sandwich Enzyme-Linked Immunosorbent Assay (ELISA) kits following the manufacturer’s protocols. Serum FOXP3 levels were measured using the Elabscience Human FOXP3 ELISA kit (Cat. No: E-EL-H1104), and serum GATA-3 levels were measured using the Elabscience Human GATA3 ELISA kit (Cat. No: E-EL-H0881) (Elabscience Biotechnology Inc., Wuhan, China). Both kits had a lower detection limit of 0.19 ng/mL, a measurement range of 0.31–20 ng/mL, and intra- and inter-assay coefficient of variation (CV) values of <10%.

Serum RORɣt levels were measured using the BT LAB Human Nuclear Receptor RORγt ELISA kit (Cat. No: E3252Hu), and serum TBX21 levels were measured using the BT LAB Human T-box Transcription Factor ELISA kit (Cat. No: E7521Hu) (Biossays Technology Laboratory, Shanghai, China). For the RORɣt kit, the lower detection limit was 3.12 ng/L with a measurement range of 7–1500 ng/L, while the intra- and inter-assay CV values were <10%. For the TBX21 kit, the lower detection limit was 0.12 ng/mL with a measurement range of 0.25–16 ng/mL; intra-assay CV was <8%, and inter-assay CV was <10%.

The ELISA procedure involved pre-coated plates with specific antibodies for each transcription factor. Serum samples and standards were added to the wells and incubated at 37 °C for the recommended time. After washing, biotin-conjugated detection antibodies and Horseradish Peroxidase (HRP) enzyme solutions were sequentially added, followed by further incubations and washes. Substrate solution was added to generate a colorimetric reaction, and the reaction was stopped using a stop solution. The absorbance values were measured at 450 nm using a microplate reader. The transcription factor concentrations were determined using standard curves and expressed as ng/mL or ng/L. In addition, a complete blood count and C-reactive protein (CRP) values were determined by standard laboratory techniques in order to exclude the possibility of infection.

### 2.4. Statistics

Statistical analyses were conducted using IBM SPSS version 27. To test the normality of data distribution, the Kolmogorov–Smirnov test was used for samples larger than 50, while the Shapiro–Wilk test was applied for samples smaller than 50. Pre- and post-treatment comparisons were performed using the Paired Sample *t*-test for normally distributed data and the Wilcoxon signed-rank test for non-normally distributed data. The correlation between variables and immune response markers was analyzed using Pearson’s correlation test (for normally distributed data) and Spearman’s rank correlation test (for non-normally distributed data). Significant correlations were further analyzed using ANOVA (for normally distributed groups) and Mann–Whitney U or Kruskal–Wallis tests (for non-parametric data). Statistical analyses were first performed on all 66 patients with both blood values and then repeated after stratifying patients according to treatment regions. A 95% confidence interval was used and *p* < 0.05 was considered statistically significant.

## 3. Results

### 3.1. Patient Characteristics

The study included 66 patients treated between September 2022 and December 2023. The demographic and clinical characteristics of the patients are summarized in Table 1. The median age was 62 years (range: 31–85 years) and the cohort consisted of 57.6% males and 42.4% females. Performance status was ECOG 0–1 in 87.9% of patients. Among the treatment regions, 22 patients (33.3%) received SBRT to the lung, 20 patients (30.3%) to the brain, and 13 patients (19.7%) to the bone. Prior to SBRT, 51 patients (77.3%) had received chemotherapy, and 5 patients (7.6%) had a history of immunotherapy. Concurrent treatments were defined as those administered within three weeks before SBRT and up to the second blood collection date. The majority of patients (65.2%) did not receive concurrent treatments, while 27.3% underwent concurrent chemotherapy and 7.5% received concurrent immunotherapy.

### 3.2. Changes in Transcription Factors

A summary of the changes in transcription factors, complete blood count and CRP levels is presented in Table 2. The levels of TBX21, a marker of Th1 cells, and GATA3, a marker of Th2 cells, significantly decreased following treatment (*p* < 0.001 and *p* = 0.005, respectively). Although the TBX21/GATA3 (Th1/Th2) ratio, a key indicator of immune activation or suppression, demonstrated a tendency to increase, this change was not statistically significant (*p* = 0.7).

Similarly, FoxP3 levels, representing immunosuppressive regulatory T cells (Tregs), exhibited a significant decline (*p* < 0.001). No significant changes were observed in RORɣt levels, which reflect Th17 cells and are considered stabilizers of Treg cells. However, a decrease was observed in the FoxP3/RORɣt (Treg/Th17) ratio, indicating a shift in immune balance towards immune activation (*p* < 0.001).

No significant differences were found in serum CRP levels or hematological parameters, including white blood cell count (WBC), absolute lymphocyte count (ALC), and absolute neutrophil count (ANC), throughout the study. These findings suggest that systemic inflammation or infection did not influence the observed changes in transcription factors.

### 3.3. Subgroup Analyses by Treatment Region

Following the completion of the analyses on the entire patient cohort, patients were divided into three treatment groups based on their primary disease sites: lung, brain, and bone. The analyses were repeated for each subgroup to evaluate region-specific changes in transcription factor levels.

In patients treated for lung tumors, significant reductions were observed in TBX21 and FoxP3 levels (*p* < 0.001 and *p* = 0.003, respectively), consistent with the overall cohort. GATA-3 levels also significantly decreased (*p* = 0.03), while RORɣt levels remained stable (*p* = 0.12). These findings suggest that SBRT in lung tumors suppresses both Th2 and Treg markers, without affecting Th17 responses.

In the brain treatment group, TBX21 and FoxP3 levels decreased significantly (*p* < 0.001 and *p* = 0.002, respectively), similar to the overall cohort. However, GATA-3 levels remained stable (*p* = 0.49), indicating that Th2 responses were not significantly altered in this subgroup. No significant changes were observed in RORɣt levels (*p* = 0.27). Interestingly, the stability of GATA-3 in the brain may suggest site-specific differences in the immune microenvironment or tumor-immune interactions.

**Figure 2:**
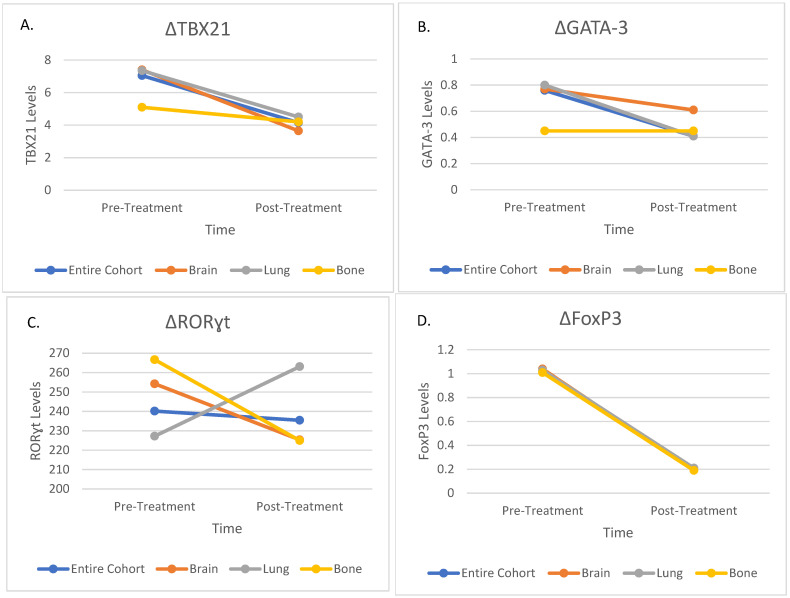
Changes in Immune Markers Before and After Treatment in the Entire Cohort and Subgroups; (**A**) Changes in TBX21 Levels; (**B**) Changes in GATA-3 Levels; (**C**) Changes in RORɣt Levels; (**D**) Changes in FoxP3 Levels. TBX21—T-box transcription factor 21; GATA-3—GATA binding protein-3; RORɣt—RAR-Related Orphan Receptor Gamma; FoxP3—Forkhead box P3.

In patients treated for bone metastases, TBX21, GATA-3, and FoxP3 levels decreased significantly (*p* = 0.02, *p* = 0.04 and *p* = 0.005, respectively), aligning with the overall cohort findings. RORɣt levels showed no significant changes (*p* = 0.48), consistent with the other treatment groups. Detailed changes in transcription factor levels for each subgroup are presented in Table 3. Visual representation of these changes is provided in Figure 2.

### 3.4. Correlation Between Transcription Factors and Clinicopathological Parameters

The relationship between patient characteristics and immune system parameters was investigated. No significant correlations were observed between demographic or clinical characteristics, including gender, smoking history, ECOG performance status, primary tumor site, histology, and changes in transcription factor levels.

The administration of chemotherapy or immunotherapies prior to or concurrently with SBRT, which differed in each patient according to the primary disease and the course of the disease, had no significant effect on transcription factor levels. These findings suggest that changes in transcription factor levels following stereotactic radiotherapy are independent of baseline clinicopathological characteristics.

### 3.5. Correlation Between Transcription Factors and Dosimetric Parameters

**Figure 3:**
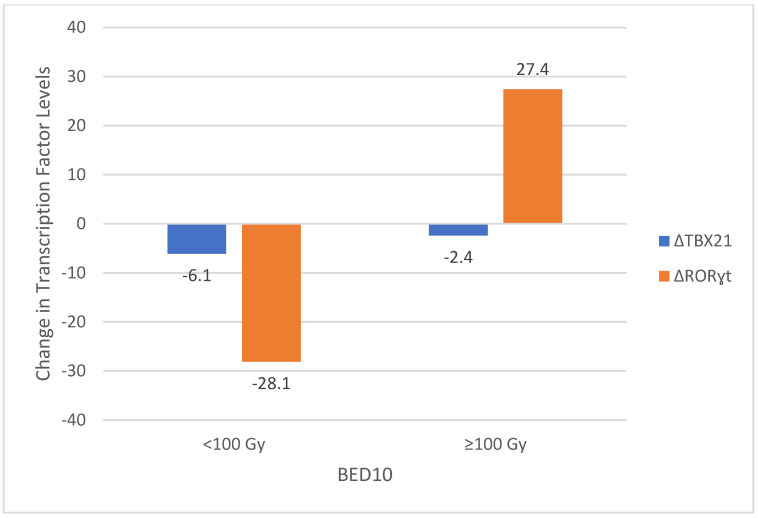
Changes in TBX21 and RORγt Levels According to BED10 Groups. TBX21—T-box transcription factor 21; RORɣt—RAR-Related Orphan Receptor Gamma; BED—Biologically Effective Dose.

The relationship between transcription factor levels and dosimetric parameters, including planning target volume (PTV) and biologically effective dose (BED10), was analyzed. In the lung treatment group, a significant correlation was observed between TBX21 levels and both PTV and BED10 values. Patients with PTV greater than 16.5 cc exhibited a smaller reduction in TBX21 levels compared to those with smaller PTVs (*p* = 0.008). Similarly, BED10 values of at least 100 Gy were associated with a smaller decrease in TBX21 levels (*p* = 0.04). Interestingly, RORɣt levels showed a significant increase when BED10 exceeded 100 Gy (*p* = 0.01), suggesting a dose-dependent activation of Th17-related pathways (Figure 3). No significant correlations were found between dosimetric parameters and changes in GATA-3 or FOXP3 levels in any treatment group.

These findings highlight the potential influence of radiation dose and target volume on Th1 and Th17 responses, particularly in lung tumors treated with higher BED10 values.

## 4. Discussion

Stereotactic radiotherapy is an advanced technique that allows for the precise delivery of a high dose of radiation directly to a tumor while minimizing radiation exposure to the surrounding healthy tissue. This is accomplished by using multiple radiation fields and achieving a rapid reduction in dose outside the target area [20,21,22]. Beyond its local tumoricidal effects, SBRT is known to trigger immune responses, making the relationship between SBRT and immune modulation an area of active research [14,23,24]. After SBRT, rapid tumor cell apoptosis releases tumor-associated antigens, which activate immature T cells via dendritic cells, ultimately leading to T cell differentiation [25,26]. Key transcription factors, including TBX21, GATA-3, RORγt and FoxP3, play a crucial role in the differentiation of T-cells into the Th1, Th2, Th17 and Treg cell types, respectively. These factors have been demonstrated to modulate the direction of immune responses, immune activation, or immune suppression [14,20,21]. Transcription factors are essential for enabling differentiated T cells to target both the primary tumor treated with radiotherapy and metastatic tumor cells located in other areas. This phenomenon, known as the abscopal effect, occurs when radiotherapy impacts not only the primary tumor site but also distant metastatic cells. The term was first introduced by Mole in 1953 [4]. Although a notable phenomenon, the abscopal effect is rarely observed in clinical practice. As a result, researchers have utilized various markers in studies for an extended period to demonstrate this immune-activating effect more objectively. The initial investigation into the relationship between SBRT and the immune response focused on analyzing T cell levels [27,28]. This was followed by studies examining CD4+ and CD8+ T cells [29,30], and later, researchers explored more specific immune markers [14,31].

In our study, we investigated the effects of SBRT on transcription factors related to immune activation and suppression, specifically TBX21 (Th1), GATA-3 (Th2), RORγt (Th17), and FoxP3 (Treg). Our findings revealed a significant decline in the levels of immunological suppressors, including GATA-3 (Th2) and FoxP3 (Treg), indicating a potential shift towards increased immune activation. Notably, TBX21 (Th1) levels exhibited variations dependent on radiation dose and volume, highlighting the potential role of radiation dosimetry in influencing immune responses. In contrast, RORγt levels, which reflect Th17 activity, mainly remained unchanged across the general cohort. However, in cases where the BED10 value exceeded 100 Gy, there was a trend towards increased RORγt levels. This suggests that higher radiation doses may activate Th17-related pathways, contributing to the overall immune response alongside Th1 cells. The involvement of Th17 responses in high-dose settings emphasizes the complex relationship between radiation dose, immune modulation, and T cell activation following SBRT.

In early animal studies, B16 melanoma tumors were implanted in mice with and without T cell deficiency. After 20 Gy of ablative radiotherapy, healthy mice showed increased T cell infiltration and significant tumor regression, while T cell-deficient mice showed no regression, suggesting the importance of T lymphocytes for the effectiveness of the radiotherapy. In another part of this study, immunocompetent mice received either 20 Gy in a single fraction or 5 Gy in four fractions. Results indicated greater antitumor immunity and tumor regression in the single-fraction group [28].

Following the demonstration of the immunomodulatory effects of stereotactic radiotherapy in numerous animal studies, clinical trials were initiated to investigate this area further. These studies explored the effects of both stereotactic radiotherapy and hypofractionated radiotherapy. The 2018 LYMPHOREC study involved 237 patients who underwent short- and long-course preoperative radiotherapy for locally advanced colorectal cancer. It aimed to evaluate the immune response in tumor tissue after radiotherapy. Researchers compared the number of tumor-infiltrating lymphocytes in pretreatment biopsy specimens with those in pathology specimens obtained surgically after preoperative radiotherapy. They observed a significant decrease in FoxP3+ T lymphocytes, along with a notable increase in the ratio of CD8+ T lymphocytes to FoxP3+ T lymphocytes following treatment [31]. In our study, we evaluated the levels of FoxP3 in the blood. Consistent with the findings from the LYMPHOREC study, we found a decrease in FoxP3 levels, indicating reduced activation of T regulatory cells (Tregs) and an increase in overall immune activation. In the present study, the investigation was confined to the study of Th cells and their transcription factors. However, due to financial reasons, an evaluation of CD8+ T cell levels following SBRT could not be conducted.

Another study conducted on 89 patients who underwent SBRT for early-stage non-small cell lung cancer (NSCLC) analyzed the levels of T cells and key transcription factors, including RORγt (Th17), TBX21 (Th1), GATA-3 (Th2), and FoxP3 (Treg), in peripheral blood samples taken before, 2 weeks, and 12 weeks after treatment. Patients were treated with SBRT at a 54–60 Gy dose in 3–8 fractions, corresponding to BED10 values exceeding 100 Gy. The study observed increased CD4+ T cells, CD8+ T cells, and all transcription factors in the second week following treatment. By the third month, GATA-3, TBX21, and RORγt levels continued to rise, while FoxP3 levels significantly decreased [14]. Similar to this study, our findings revealed a significant reduction in FoxP3 levels, indicative of decreased Treg activity. Unlike the findings of this study, where GATA-3 levels continued to rise following treatment, we observed a significant reduction in GATA-3 levels, suggesting a suppression of Th2 activity. The observed difference may be attributable to tumor characteristics, patient demographics, and radiation dosimetry variations across the two studies. Interestingly, in our study, we observed a non-significant increase in RORγt levels specifically in lung cancer patients, consistent with the observations in the aforementioned study where all patients had lung cancer. This parallel finding may be partially explained by the high BED10 values (>100 Gy) used in both studies, which appear to activate Th17 pathways, as reflected by the increase in RORγt levels. However, it is worth noting that the previous study did not report data on PTV, which could have influenced the observed immune activation. Given the dose- and volume-dependent changes in TBX21 levels in our study, it is plausible that larger PTV may have also contributed to the observed immune responses in their study.

In the literature, it has been shown that administering immunotherapy—especially CTLA-4 and PD-L1 inhibitors—concurrently with SBRT can enhance the anti-tumor immune response. However, the current study did not find a relationship between the use of immunotherapy and these transcription factors, likely due to the limited number of patients who received immunotherapy.

It is important to acknowledge that this study has certain limitations. The relatively small sample size, both for the overall cohort and within subgroup analyses, limited the interpretation of results, particularly for patients treated in the liver, adrenal, pancreas, and prostate regions. Similarly, the number of patients receiving concurrent treatments, especially immunotherapy, was insufficient to demonstrate the anticipated synergistic effects. Additionally, due to financial constraints, our analysis focused solely on CD4+ T cell subsets, preventing us from evaluating CD8+ T cell responses. The small sample size, short follow-up period, and heterogeneity in primary tumor regions, tumor histologies, treatment sites, and prior treatment histories limited our ability to conduct survival analyses and local control evaluations to assess the clinical implications of our findings. Despite these constraints, our study provides valuable insights into the immunomodulatory effects of SBRT. Future research that includes larger cohorts, comprehensive immune profiling, and extended follow-up is essential to validate and expand upon these findings.

## 5. Conclusions

In conclusion, our study demonstrates that SBRT has significant immunomodulatory effects, characterized by a reduction in immunosuppressive markers such as FoxP3 (Treg) and GATA-3 (Th2). Additionally, we observed dose- and volume-dependent trends in TBX21 (Th1) levels and a tendency for RORγt (Th17) levels to increase in high-dose settings. These findings suggest that SBRT not only reduces immunosuppression but also has the potential to stimulate pro-inflammatory immune responses, particularly through Th1 and Th17 pathways.

Given the growing evidence of synergy between radiotherapy and immune checkpoint inhibitors, combining SBRT with immunotherapy may amplify systemic anti-tumor immunity and improve treatment outcomes. Further research is required to optimize treatment protocols, including dose delivery, target volume, and sequencing strategies, to fully harness the clinical potential of SBRT-induced immune activation.

## Figures and Tables

**Table 1:** Baseline Demographic and Treatment Characteristics of the Patients.

Parameter		Value
Age, y, median (range)		62 (31–85)
Sex, male/female		38/28 (%57.6/%42.4)
ECOG Performance Score	0	18 (%27.3)
	1	40 (%60.6)
	2	8 (%12.1)
Smoke	No	35 (%53)
	Yes	31 (%47)
Primary Disease Site and Histology	Lung	29 (%43.9)
	Adenocarcinoma	20 (%30.2)
	SCC	5 (%7.6)
	Small Cell Carcinoma	4 (%6.1)
	Brain (Glioblastoma Multiforme)	2 (%3)
	Breast	9 (%13.6)
	Head and Neck	7 (%10.6)
	SCC	1 (%1.5)
	Glomus	5 (%7.6)
	Schwannoma	1 (%1.5)
	Genitourinary System	8 (%12.1)
	Renal Cell Carcinoma	5 (%7.6)
	Prostate Adenocarcinoma	2 (%3)
	Urothelial Carcinoma	1 (%1.5)
	Gastrointestinal System	5 (%7.6)
	Colorectal Adenocarcinoma	4 (%6.1)
	Pancreatic Adenocarcinoma	1 (%1.5)
	Endometrial Adenocarcinoma	1 (%1.5)
	Soft Tissue Sarcoma	2 (%3)
	Kordoma	2 (%3)
	Peritoneal Mezothelioma	1 (%1.5)
SBRT Site	Lung	22 (%33.3)
	Brain	20 (%30.3)
	Bone	13 (%19.7)
	Neck	6 (%9.1)
	Surrenal	2 (%3)
	Liver	1 (%1.5)
	Pancreas	1 (%1.5)
	Prostate	1 (%1.5)
Previous Chemotherapy	No	15 (%22.7)
	Yes	51 (%77.3)
Previous Immunotherapy	No	61 (%92.4)
	Yes	5 (%7.6)
Previous Radiotherapy	No	34 (%51.5)
	Yes	32 (%48.5)
Chemotherapy after SBRT	No	39 (%59.1)
	Yes	27 (%40.9)
Immunotherapy after SBRT	No	59 (%89.4)
	Yes	7 (%10.6)
Radiotherapy after SBRT	No	48 (%72.7)
	Yes	18 (%27.3)
Concomitant Treatment with SBRT	No	43 (%65.2)
	Yes	23 (%34.8)
	Chemotherapy	18 (%27.3)
	Immunotherapy	5 (%7.5)

ECOG—Eastern Cooperative Oncology Group; SCC—Squamous Cell Carcinoma; SBRT—Stereotactic Body Radiotherapy.

**Table 2:** Pre- and Post-Treatment Changes in Immune Markers.

Parameter	Pre-Treatment Levels	Post-Treatment Levels	*p* Value
TBX21	7.05 (2.3–16)	4.15 (1.8–16)	***p* < 0.001**
GATA 3	0.76 (0.19–20)	0.41 (0.19–19.83)	***p* = 0.005**
TBX21/GATA3	8.89 (0.8–84.21)	11.46 (0.15–84.21)	*p* = 0.76
RORɣt	240.15 (115.9–1500)	235.45 (169.7–1500)	*p* = 0.49
FoxP3	1.03 (0.19–3.08)	0.19 (0.19–11.44)	***p* < 0.001**
FoxP3/RORɣt	0.004 (0–0.02)	0.001 (0–0.05)	***p* < 0.001**
WBC	6.83 (2–15.5)	7.08 (2.2–18.8)	*p* = 0.51
ALC	1.46 (0.4–2.9)	1.36 (0.4–3)	*p* = 0.092
ANC	4.80 (1–14.2)	4.92 (0.9–17)	*p* = 0.74
CRP	4.83 (1.37–81.5)	6.45 (1.53–80)	*p* = 0.143

TBX21—T-box transcription factor 21; GATA-3—GATA binding protein-3; RORɣt—RAR-Related Orphan Receptor Gamma; FoxP3—Forkhead box P3; WBC—white blood cells; ALC—absolute lymphocyte count; ANC—absolute neutrophil count; CRP—C-reactive protein. Statistically significant values are indicated by bold font.

**Table 3:** Subgroup Analysis of Transcription Factor Levels by Treatment Regions.

Parameter	Region	Pre-Treatment Levels	Post-Treatment Levels	*p* Value
TBX21	Brain	7.4 (2.3–16)	3.65 (1.8–16)	***p* < 0.001**
	Lung	7.35 (5.3–16)	4.5 (2.4–16)	***p* < 0.001**
	Bone	5.1 (2.4–16)	4.2 (2.2–16)	***p* = 0.021**
GATA-3	Brain	0.77 (0.19–8.34)	0.61 (0.19–9.62)	*p* = 0.49
	Lung	0.80 (0.19–2.13)	0.41 (0.19–17.61)	***p* = 0.03**
	Bone	0.45 (0.19–1.62)	0.35 (0.19–1.02)	***p* = 0.047**
RORɣt	Brain	254.25 (146.1–1500)	225.35 (169.7–1500)	*p* = 0.27
	Lung	227.25 (154.7–1500)	263.15 (171.2–1500)	*p* = 0.12
	Bone	266.7 (115.9–1500)	225 (170.2–1500)	*p* = 0.48
FoxP3	Brain	1.04 (0.19–2.16)	0.19 (0.19–0.93)	***p* = 0.002**
	Lung	1.03 (0.19–1.82)	0.21 (0.19–11.44)	***p* = 0.003**
	Bone	1.01 (0.19–1.13)	0.19 (0.19–0.48)	***p* = 0.005**

TBX21—T-box transcription factor 21; GATA-3—GATA binding protein-3; RORɣt—RAR-Related Orphan Receptor Gamma; FoxP3—Forkhead box P3. Statistically significant values are indicated by bold font.

## Data Availability

Additional data supporting the reported results can be requested from the corresponding authors.
